# Comparison of Metabolic Alterations in Serum and Milk Whey Between Inactive Ovaries and Estrus Dairy Cows

**DOI:** 10.3389/fvets.2020.609391

**Published:** 2021-01-14

**Authors:** Chang Zhao, Yunlong Bai, Shixin Fu, Ling Wu, Cheng Xia, Chuang Xu

**Affiliations:** ^1^College of Animal Science and Veterinary Medicine, Heilongjiang Bayi Agricultural University, Daqing, China; ^2^Heilongjiang Provincial Technology Innovation Center for Bovine Disease Control and Prevention, Daqing, China

**Keywords:** dairy cows, inactive ovaries, differential metabolites, serum, milk whey

## Abstract

Inactive ovaries (IOs) affect the estrus cycle and timed artificial insemination (TAI) efficiency in dairy cows during early lactation. The objective of the experiment was to determine metabolic changes in the serum and milk whey of dairy cows with IO and estrus. Twenty-eight healthy postpartum Holstein cows in similar age, milk production, and body condition were selected at 30 days postpartum for tracking to 70 days postpartum, and estrus performance was recorded through Afi Farm® software. The ovarian status and follicular diameter of dairy cows were examined by an experienced breeder through B-ultrasound and rectal examination. Fourteen normal estrus cows were allocated to control group A and 14 cows with IO to group B, all at 30–70 days postpartum. The serum and milk whey in the two groups of cows at 70 days postpartum were used for non-targeted nuclear magnetic resonance (^1^H-NMR) analysis to measure the different metabolites of cows with IO. In group B compared with group A at 70 days postpartum, there was an increase in the milk whey of six different metabolites including succinate, creatine phosphate, glycine, myo-inositol, glycolate, and orotate and a decrease in the milk whey of seven metabolites, including alanine, creatinine, o-phosphorylcholine, lactose, taurine, galactose, and glucose-1-phosphate. There was an increase in the serum of group B cows of four differential metabolites, including 3-hydroxybutyrate, acetate, glutamine, and glycine and a decrease in the serum of nine differential metabolites, including alanine, succinate, citrate, creatinine, o-phosphocholine, glucose, myo-inositol, tyrosine, and histidine compared with group A. Group B cows with IO had decreased glucose metabolism and impaired tricarboxylic acid cycle, increased lipid mobilization, and abnormal amino acid metabolism. The study provides a potential prevention strategy for IO in dairy cows in future.

## Introduction

At present, there is a poor reproduction in dairy cows with high milk yield in intensive cattle farms in many countries (1). The genetic selection of high-yield dairy cows leads to the diversion of available nutrients toward milk synthesis and less to fertility and physiological processes (2). As a result, the reproductive efficiency of dairy cows is reduced after birth, and the days to first ovulation and conception after delivery are prolonged (3). In the past decades, the first insemination and conception rates of dairy cows in the United Kingdom and the United States declined at an annual rate of 0.5 (4) and 1% (5). Postpartum reproductive disorders in dairy cows seriously affect the economical profit of dairy farms, becoming a limiting factor restricting the development of the dairy industry worldwide. Inactive ovaries (IO) are a type of postpartum anestrus in dairy cows caused by the temporary disturbance of ovarian function and a lack of periodic follicular activity (6), with an incidence of 26.3–50% of anestrus in dairy cows (7, 8). Matthew (2011) proposed that body metabolism affects follicular growth, especially the metabolism of estradiol, growth hormone, insulin-like growth factor-I, insulin,alanine, and glutamine (9). Previous studies showed that postpartum IO in dairy cows was mainly due to the imbalance of nutrient distribution caused by high lactation and negative energy balance (NEB) after calving. The current research of IO in dairy cows is mainly focused on the effects of certain substances on follicular growth or ovarian activity (10–13). Our previous research found that, compared with estrus cows, serum metabolic profiles of IO cows may be altered in postpartum energy metabolism to affect follicular growth (14–16). In dairy cows, milk whey is more readily available than serum and can also reflect the metabolic changes, so these disease biomarkers would be more suitable for detecting a dairy cow's health. Currently, there is a lack of research on the changes in the overall metabolites of the serum and whey in IO and healthy cows. This study hypothesizes that whey metabolism may be similar to serum metabolism of dairy cows with IO.

It has been known that metabolites are the most direct and comprehensive reflection of the metabolic state of an organism (17). Metabolomics has been widely used to quantitatively study all compounds or metabolites produced by cells and tissues under normal and diseased conditions (18). Nuclear magnetic resonance (^1^H-NMR) is a metabolomics technology that has advantages of simple sample pretreatment, no sample deviation, and no damage in the analysis process (19). The use of ^1^H-NMR and liquid chromatography quadruple time-of-flight mass spectrometry (LC/MS) or gas chromatography/mass spectrometry (GC/MS) have different advantages and disadvantages. Because the data set obtained by ^1^H-NMR is large, it also contains a lot of interference and noise and other information. The ^1^H-NMR raw data require preprocessing, including noise filtering, peak matching, normalization, and standardization to make it a data form suitable for multivariate statistical analysis (20), so most other analytical techniques are inherently more sensitive than ^1^H-NMR, with lower limits of detection typically being 10–100 times better (21). However, ^1^H-NMR technology requires less pretreatment of the sample for analysis and has a high level of reproducibility and quantitative ability and is useful in identifying unknown metabolites (18). In the analysis of body fluids, the supernatant is usually centrifuged, and only buffer or water is added to control the pH and viscosity (22).

Plasma metabolic profiles of dairy cows based on ^1^H-NMR have been used to obtain biomarkers or to explore the metabolic mechanism of ketosis, mastitis, inactive ovaries, and other diseases (23–26). Wei Xu used ^1^H-NMR to identify 14 compounds in whey (27). Timothy DW used ^1^H-NMR to identify 15 differential metabolites in 707 cows serum (28), and we have previously used ^1^H-NMR to identify 32 compounds in the serum of ovarian quiescence cows (1). Milk should be used to screen biomarker or metabolic alteration of ovary diseases because of its convenience (29). To date, no comparison has been found between the serum and milk whey metabolic profile of postpartum IO in dairy cows. In this study, non-targeted metabolomics with the assistance of ^1^H-NMR was used to detect milk whey and serum metabolomics of dairy cows with IO to further explore the pathogenesis and potential prevention strategy for IO in dairy cows.

## Materials and Methods

### Animals

The study was administrated in strict accordance with the Guide for the Care and Use of Laboratory Animals of the National Institutes of Health. All experiments on animals were carried out according to the standards approved by the Animal Welfare and Research Ethics Committee at Heilongjiang Bayi Agricultural University.

The experiment was carried out in an intensive dairy farm with 1,500 Holstein dairy cows. The total mixed ration of tested dairy cows complied with the Nutrient Requirements of Dairy Cattle (NRC) 2001 feeding standard of dairy cows and consisted of 55.60% dry matter (DM), 16% crude protein, 5.60% fat, 180 g calcium (Ca), 116 g phosphorus (P), 39.10% neutral detergent fiber, 20.30% acidic detergent fiber, and 1.75 mcal/DM lactation net energy.

Twenty-eight healthy postpartum Holstein cows aged 3–4 years with a body condition score of 3.0–3.50 and a daily milk production of 33–35 kg/day were selected at 30 days postpartum for tracking to 70 days postpartum, and cow background information and estrus performance were recorded using Afi Farm® software. It is ensured that there is no difference in body condition score (BCS), BCS loss, daily milk production between the two groups of cows within 30–70 days after delivery, and there are no other diseases. The ovarian status and follicular development of dairy cows were examined by an experienced breeder using B-ultrasound and rectal examination. Fourteen normal estrus cows were allocated to control group A, and 14 cows with IO were in group B at 70 days postpartum. Selection criteria for group A cows were spontaneous estrus with obvious estrus symptoms, normal uterus, and 15–20 mm ovarian follicles at ovulation. Selection criteria for group B cows with IO were lack of follicular development on either ovary and the follicle diameter was <8 mm, follicle diameter increase of <2 mm in 2 days, and there was no corpus lutea on the ovaries (14, 15).

### Sample Collection

A 10-ml blood sample at 70 days postpartum was collected from the coccygeal vein in two groups of cows in the early morning before feeding and immediately centrifuged at 3,000 rpm for 10 min and then at 12,000 rpm for 10 min, with the serum being placed in a 1.5-ml tube, frozen in liquid nitrogen, and then stored in −80°C for ^1^H-NMR testing. Milk sample collection was performed at the same time of blood sample collection in two groups of cows. The samples were then stored at −80°C for the ^1^H-NMR test.

### ^1^H-NMR Analysis

The sample handling was consistent with the ^1^H-NMR sample handling method reported earlier (1, 24, 27). Serum samples were adjusted to 99.8% (v/v) D_2_O phosphate buffer solution containing 0.05% (w/v) total suspended particulate (TSP matter: 0.2 M Na_2_HPO, 0.2 M NaH_2_PO_4_, NaH_2_PO_4_) at pH of 7.0 and centrifuged at 12,000 × g for 20 min at 4°C, and the supernatant was collected into a 5-mm nuclear magnetic tube for testing. Milk whey samples from two groups of cows at 70 days postpartum were prepared using 2 ml of whey in each tube in a 10-mL centrifuge tube. Two milliliters of methanol and 2 ml acetonitrile were added to each sample, mixed and allowed to stand at −20°C for 20 min to separate fully. The remaining steps were the same as for serum samples (22, 30, 31).

For ^1^H-NMR analysis, the above processed serum and milk whey samples were performed on a 500-MHz NMR spectrometer (AVANCE III, Bruker, Switzerland). The test temperature was 298 K, and D_2_O and TSP are used for field locked and chemical shift reference (^1^H, 0.00 ppm), respectively. The pulse sequence used a transversely relaxed Carr–Purcell Meiboom–Gill (CPMG) sequence [90 (τ-180–τ) n-acquisition] and a total spin echo delay of 10 ms (2 nτ). The number of ^1^H-NMR acquisition scans (NS) was 32, the number of sampling points (TD) was 32 k, and the spectral width was 10,000 Hz.

All ^1^H-NMR data for serum and milk whey samples was corrected for zero, phase, and baseline in the Topspin software, and the peak position of the internal reference TSP was adjusted to zero displacement. The processed spectra were imported into the R software and again corrected for zero, baseline, and phase. For serum sample spectra, 0.015 ppm units for uniform interval integration was used within the 0.8–8.5 ppm chemical shift interval to reduce the number of data points while removing the water peak and resonance in the affected region from 4.5 to 5.18 ppm signal. For whey samples, the integration range was 0.75–8.4 ppm, and the water peak, and its affected area, was 4.4–5.175 ppm. All spectrum data were normalized by probability quotient normalization (PQN), followed by Pareto averaged centering and scaling.

The compounds were identified using Chenomx software (Chenomx, Canada) to fit and compare the peaks, select compounds with good peak shape matches, and combine the Human Metabolome Database (HMDB) and the Madison Metabolomics Consortium Database (MMCD). These were then analyzed with statistical total correlation spectroscopy (STOCSY). Method auxiliary identification of peak metabolites was performed on ^1^H-NMR spectra. All integrated data were normalized and integrated to perform multivariate statistical analysis as described by Zhang et al. (1) and Hongyou et al. (32).

### Multivariate Statistical Analysis

SIMCA-P10.0 (Sweden, Umetrics AB, Umeå) was used to perform multivariate statistical analysis on the data, including partial least squares discriminant analysis (PLS-DA) and orthogonal signal correction (OSC-PLS-DA). Principal component analysis of plasma metabolite ^1^H-NMR signals, analysis and comparison of the overall presentation of sample distributions, judgment of differences between groups, and determination of the principal components were conducted. Through OSC-PLS-DA analysis of the correlation coefficient of each metabolite, statistically significant metabolites were further summarized.

Combined with a one-way analysis of variance (ANOVA), 0.05 was set as the threshold for screening differentially expressed metabolites. Differential metabolites causing differences between groups were obtained. The KEGG (https://www.kegg.jp) database was used to search the metabolic pathways related to the differential metabolites.

## Results

### Identification of Differential Metabolites

**Figure 1:**
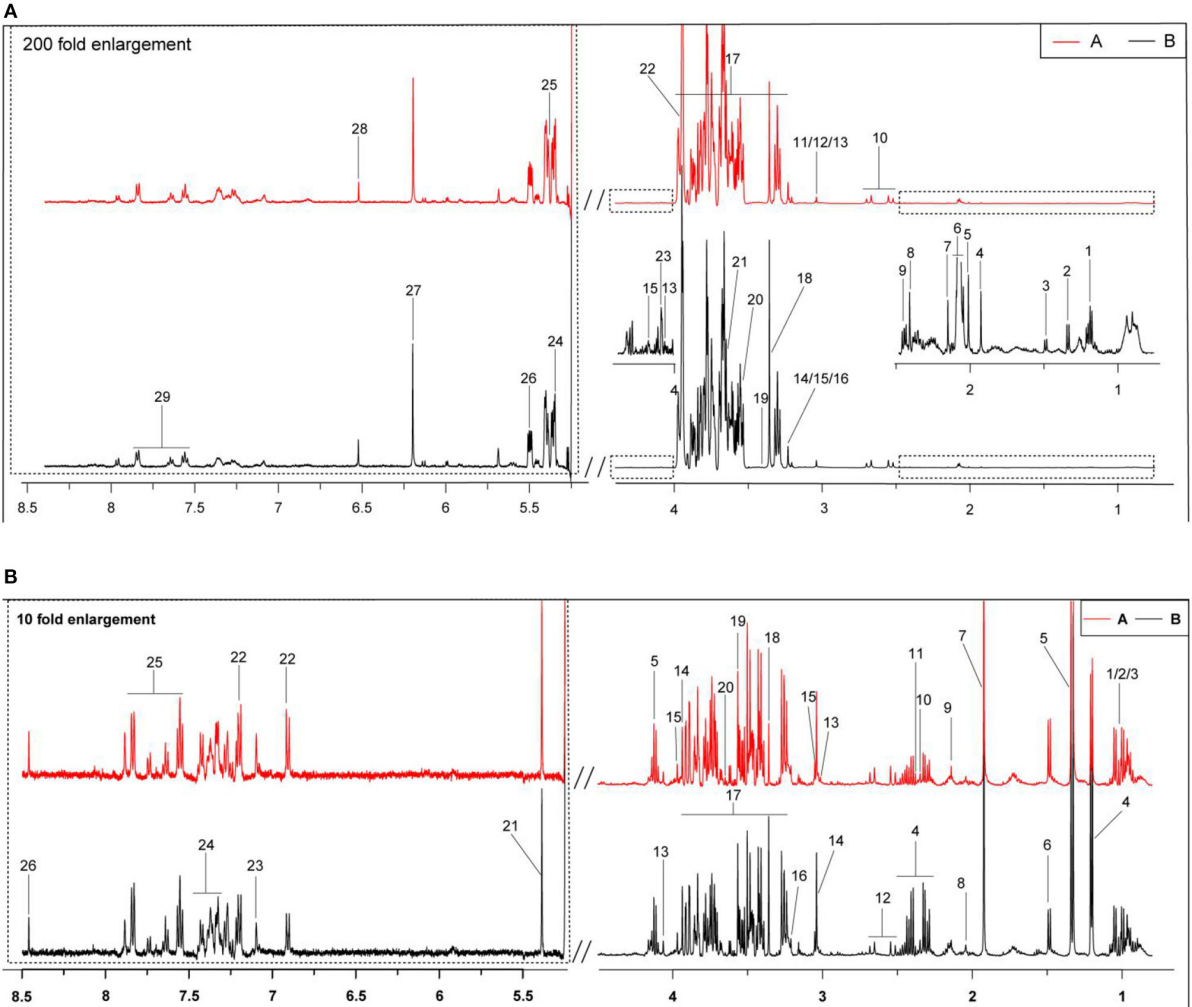
Typical ^1^H-NMR spectra (500 Hz) of milk whey and serum from groups A and B. (A) Estrus group (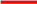); (B) inactive ovaries group (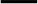). **(A)** The typical ^1^H-NMR spectra of milk whey. 1, 3-hydroxybutyrate; 2, lactate; 3, alanine; 4, acetate; 5, acetamide; 6, N-acetyl carbohydrates; 7, acetylcholine; 8: succinate; 9, 2-oxoglutarate; 10, citrate; 11, creatine; 12, creatine phosphate; 13, creatinine; 14, choline; 15, O-phosphocholine; 16, glycerophosphocholine; 17, lactose; 18, methanol; 19, taurine; 20, glycine; 21, myo-Inositol; 22, glycolate; 23, galactose; 24, maltose; 25, allantoin; 26, glucose-1-phosphate; 27, orotate; 28, fumarate; 29, hippurate. **(B)** The typical serum ^1^H-NMR spectra. 1, Isoleucine; 2, leucine; 3, valine; 4, 3-hydroxybutyrate; 5, lactate; 6, alanine; 7, acetate; 8, glutamate; 9, glutamine; 10, pyruvate; 11, succinate; 12, citrate; 13, creatinine; 14, creatine; 15, creatine phosphate; 16, phosphocholine; 17, glucose; 18, methanol; 19, glycine; 20, myo-inositol; 21, allantoin; 22, tyrosine; 23, phenylalanine; 24, hippurate; 25, histidine; and 26, formate.

In Figures 1A,B are typical ^1^H-NMR spectra of the milk whey and serum from the two groups. All the signal peaks were in the range of 0.5–8.5 ppm, excluding the 4.4–5.175 ppm water peak and its affected area. The 5.175–8.5 ppm in the low field area was expanded 200 and 10 times for the observation of milk whey and serum, and 29 and 26 compounds from the milk whey and serum were identified, respectively between the two groups.

**Figure 2:**
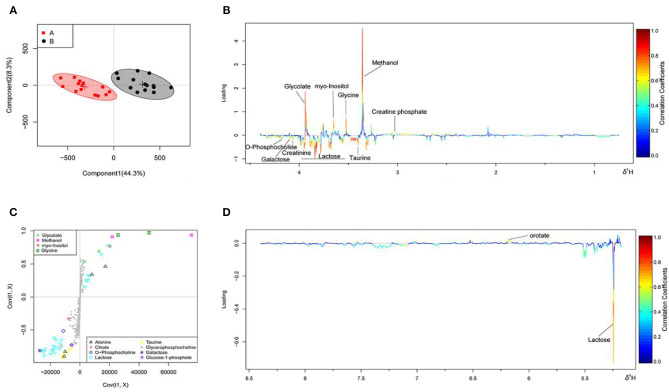
Orthogonal signal correction partial least squares discriminant analysis (OSC-PLS-DA) of milk whey ^1^H-NMR data from groups A and B. **(A)** Score plot in which one point represents one sample and one ellipse represents a confidence interval of 95%. A: estrus group (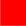); B: inactive ovaries group (•). **(B,D)** Corresponding loadings plot (0.5–4.4 ppm, 5.175–8.5 ppm) where the significance is from highest in red to lowest in blue. Metabolites above the baseline are more abundant in group B, while under the baseline are more abundant in group A. **(C)** S-plot in which different metabolites are distinguished by different colors and shapes. The different metabolites are in the upper right or lower left, and the metabolite is more significantly different if it is farther away from the origin.

**Figure 3:**
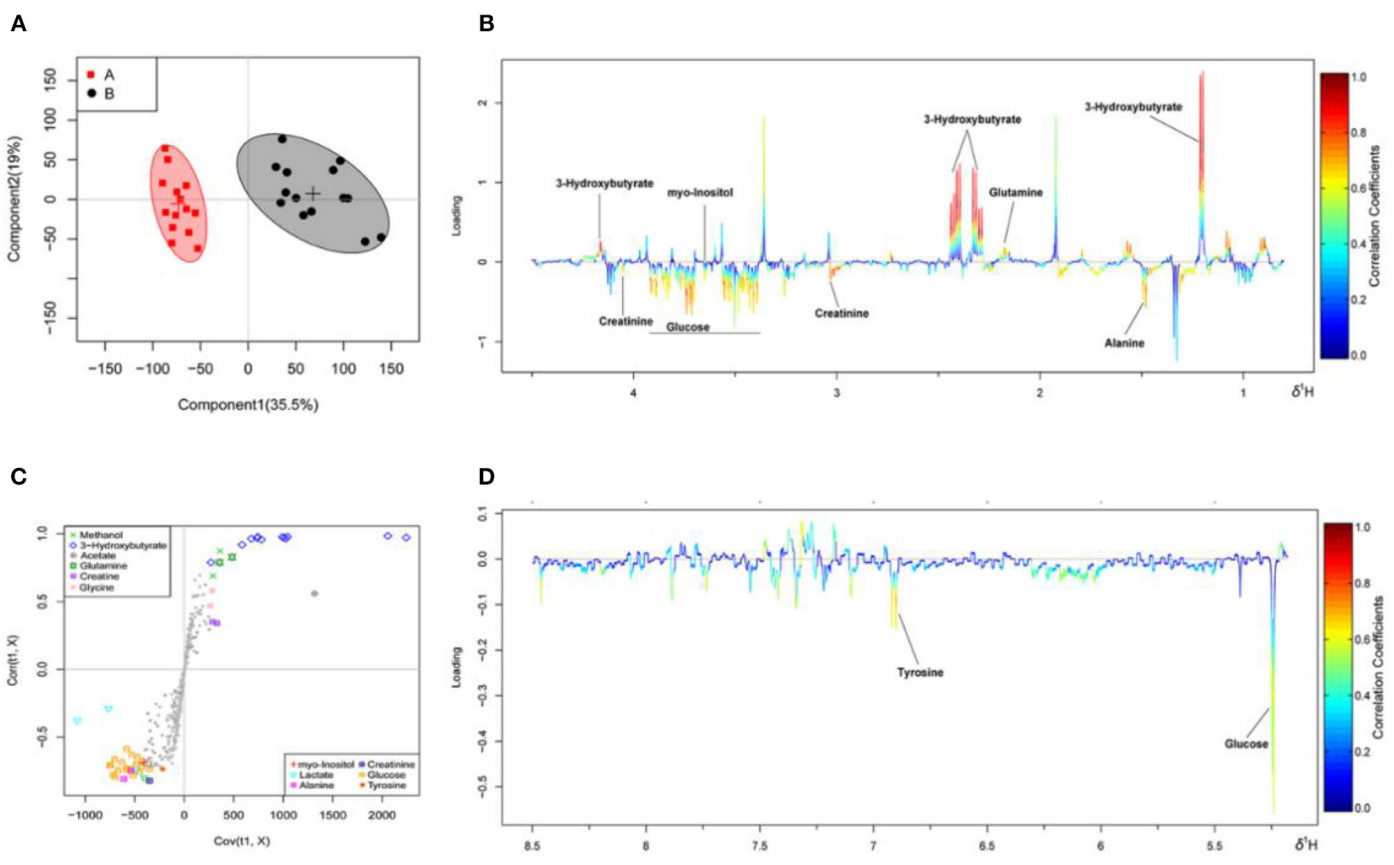
Orthogonal signal correction partial least squares discriminant analysis (OSC-PLS-DA) of serum ^1^H-NMR data from groups A and B. **(A)** Score plot in which one point represents one sample and one ellipse represents a confidence interval of 95. A: estrus group (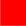); B: inactive ovaries group (•). **(B,D)** Corresponding loadings plot (0.5–4.4 ppm, 5.175–8.5 ppm) where the significance is from highest in red to lowest in blue. Metabolites above the baseline are more abundant in group B, while under the baseline are more abundant in group A. **(C)** S-plot in which different metabolites are distinguished by different colors and shapes. The different metabolites are in the upper right or lower left, and the metabolite is more significantly different if it is farther away from the origin.

The OSC-PLS-DA analysis charts of the milk whey and serum ^1^H-NMR data of groups A and B are shown in Figures 2, 3 by multivariate statistical analysis. In the OSC-PLS-DA score plot in Figures 2A, 3B, the two groups of cows were significantly separated on the left (red square) and right sides (black dot), which were not overlapped between the groups. For the first principal component (PC1) in the score map, the corresponding color S-plot in Figures 2C, 3C and the color loadings plot in Figures 2B,D, 3B,D were obtained to reveal the differential metabolites in the milk whey and serum of group B. By combining the OSC-PLS-DA score plot, color S-plot, and color loadings plot, 10 different metabolites in the milk whey were obtained in groups A and B. Compared with group A, the elevated metabolites in the milk whey samples of group B were glycolate, inositol, glycine, creatine phosphate, and orotate, and the decreased metabolites were lactose, taurine, creatinine, galactose, and phosphocholine. Methanol differences due to unclean volatilization during sample processing was excluded. Similarly, combining the OSC-PLS-DA score plot, color S-plot, and color loadings plot, seven different metabolites were determined in the serum of groups A and B. Compared with group A, an increase in β-hydroxybutyric acid (BHBA), inositol, and glutamine, glucose, alanine and creatinine, and a decrease in tyrosine in the serum of group B.

In Table 1, metabolites in the milk whey and serum of groups A and B were analyzed using multivariate statistical analysis and univariate analysis. Thirteen different metabolites were identified in milk whey. Compared with group A, milk whey, succinate, creatine phosphate, glycine, inositol, glycolate, and orotate in group B were significantly increased, while in group B, alanine, creatinine, phosphorylcholine, lactose, taurine, galactose, and glucose-1-phosphate were significantly reduced. There were 13 differential metabolites in the serum between group A and B. The levels of β-hydroxybutyric acid, acetate, glutamine, and glycine were significantly increased in the serum of group B, and alanine, succinate, citrate, creatinine, phosphorylcholine, glucose, inositol, tyrosine, and histidine were significantly decreased in the serum of group B. It is worth noting that alanine, creatinine, and O-phosphocholine were decreased in the serum and whey of group B and glycine was elevated, while succinate showed opposite results in the serum and whey of group B.

**Table 1:** Metabolites in the milk whey and serum of dairy cows with inactive ovaries (IO) and estrus.

**Whey metabolites**	**log2(FC)**	***P***	**Serum metabolites**	**log2(FC)**	***P***
Galactose	−0.74	^***^	3-Hydroxybutyrate	1.01	^***^
Alanine	−0.45	^*^	Alanine	−0.35	^***^
Succinate	0.59	^**^	Acetate	0.34	^*^
Creatine phosphate	0.23	^*^	Glutamine	0.34	^**^
Creatinine	−0.47	^**^	Succinate	−0.33	^*^
O-Phosphocholine	−1.03	^*^	Citrate	−0.26	^*^
Lactose	−0.63	^**^	Creatinine	−0.18	^**^
Taurine	−1.50	^***^	O-Phosphocholine	−0.38	^*^
Glycine	0.35	^***^	Glucose	−0.26	^**^
myo-Inositol	0.16	^*^	Glycine	0.18	^*^
Glycolate	0.25	^*^	myo-Inositol	−1.18	^**^
Orotate	0.24	^*^	Tyrosine	−0.15	^*^
Glucose-1-phosphate	−0.88	^*^	Histidine	−0.29	^**^

*If log2(FC) is a negative number, it means that the compound is downregulated in group B compared to group A. If log2(FC) is a positive number, it means that the compound is upregulated in group B compared to group A*.

* *P < 0.05*.

** *P < 0.01*.

*** *P < 0.001*.

### Differential Metabolite Pathway Analysis

**Figure 4:**
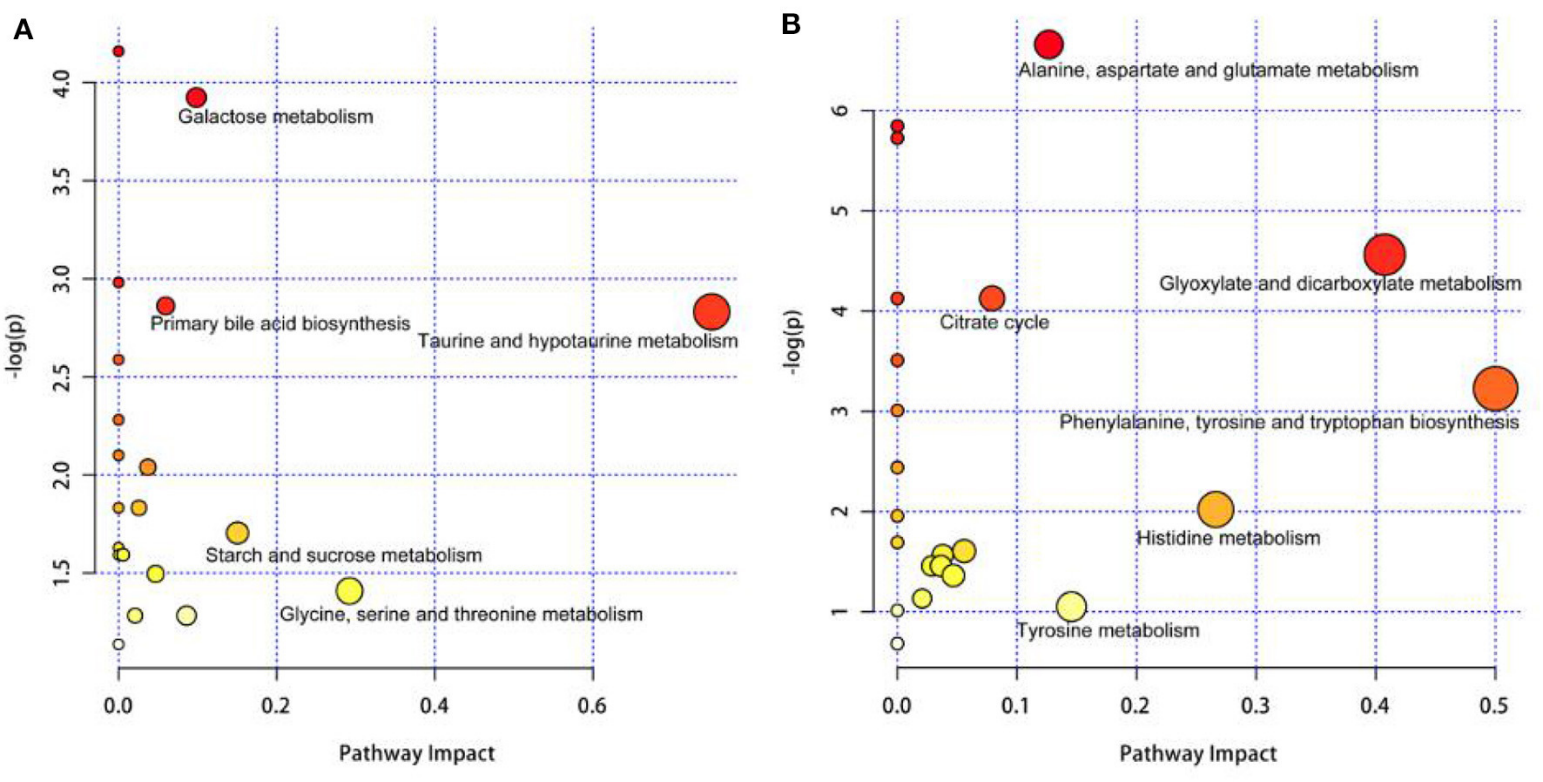
Pathway analysis of the differential metabolites in the milk whey and serum of group B cows. Bubble area is proportional to the impact of each pathway, with color denoting the significance from highest in red to lowest in white. **(A)** Pathway analysis of the differential metabolites in the milk whey. **(B)** Pathway analysis of the differential metabolites in the serum.

In Figure 4A, the differential metabolites in the milk whey of group B cows were mainly involved in the metabolism of taurine and hypotaurine, galactose metabolism, and primary bile acid biosynthesis. In Figure 4B, the differential metabolites in the milk whey of group B cows were mainly involved in alanine, aspartate, and glutamate metabolism, glyoxylate and dicarboxylic acid metabolism, citric acid cycle, and phenylalanine, tyrosine, and tryptophan organism synthesis. Both milk whey and serum have a common glucose metabolism pathway, while serum is primarily an amino acid metabolic pathway.

## Discussion

**Figure 5:**
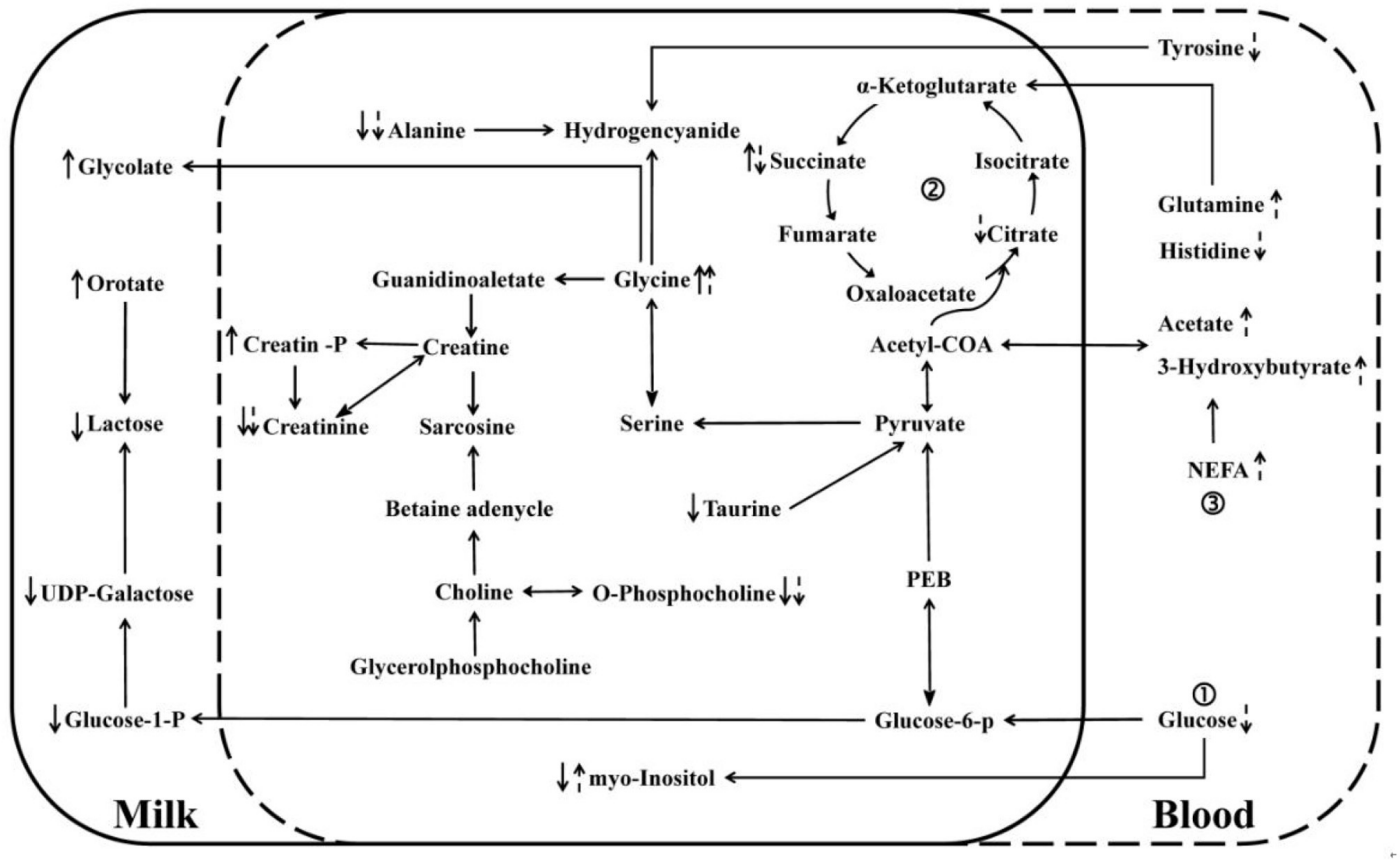
Network interaction diagram of differential metabolites in whey and serum. “↑” and “↓” denote an increase and decrease, respectively. Solid arrows indicate changes in milk whey, and dashed arrows indicate changes in serum glucose metabolism, trichloroacetic acid (TCA) cycle, and lipid metabolism.

From the database search results of metabolites in Kyoto Encyclopedia of Genes and Genomes (KEGG), linking the differential metabolites in milk with the differential metabolites in blood, a metabolic interaction network diagram (Figure 5) was developed to clearly reveal the relationship between differential metabolites and inactive ovaries. The main metabolic alteration in the milk whey and serum of dairy cows with IO was the weakening of the glucose metabolism process and the trichloroacetic acid (TCA) process and the enhancement of lipid mobilization and abnormal amino acid metabolism.

### The Effect of Glucose Metabolism on Group B Cows

First of all, the experiment found that the serum level of glucose, tyrosine, histidine, alanine, succinate, and citrate was decreased in dairy cows with IO. It has been reported that tyrosine, histidine, and alanine as glycogenic amino acids usually supply energy directly by TCA in mammals (33) and can also be converted into glucose (Glu) for energy supply by gluconeogenesis in the liver of dairy cows under negative energy balance (34). Succinate and citrate are the main members of TCA that maintain energy supply by β-oxidation in animals (35). In addition, glucose is an important energy source for follicular development (36). When the energy supply through TCA was reduced by the lower serum succinate and citrate level in dairy cows, the follicles could not be provided with sufficient energy, making the nicotinamide adenine dinucleotide phosphate (NADPH) hydrogen donor insufficient and resulting in reduced sex hormone synthesis leading to poor follicular development (37). This study indicated that dairy cows with IO had insufficient energy due to the weak gluconeogenesis of glycogenic amino acids and the decreased TCA function of succinate and citrate.

Second, the milk whey levels of lactose, galactose, and glucose-1-phosphate were significantly decreased in dairy cows with IO, consistent with that in the serum and indicating that cows with IO were in NEB due to the lack of glucose and other disaccharides in the serum or milk whey. Lactose is the main sugar in milk of dairy cows. Studies have shown that about 80% of glucose in the mammary gland is used for lactose synthesis (38, 39). Compared with other organs, the uptake of glucose in the mammary gland is not affected by insulin but flows to the mammary gland by reducing insulin sensitivity in skeletal muscle and adipose tissue (40).

Finally, the results showed some metabolic alteration of amino acid in the milk whey of dairy cows with IO. Alanine is a sugar-producing amino acid that may indirectly supply energy (41). Its decrease in the milk whey of dairy cows with IO indicated that the ability of alanine to supply the energy demand is limited. Glycine is a simple non-essential amino acid that also is a glycogenic amino acid (42). Its increase in the whey of dairy cows with IO is beneficial to alleviate the energy status of dairy cows with IO through gluconeogenesis.

The metabolomics of the serum and whey suggested that glucose metabolism plays a key role in the development of ovarian follicles or inactive ovaries in dairy cows during early lactation. This is consistent with the experimental results of Saumel et al. (43) supplementing glucose in hamster ovary cells.

### Effect of Lipid Metabolism on Dairy Cows With IO

Taurine is mainly synthesized by the cysteine sulfinate pathway to promote the removal of cholesterol in the liver (44). It may reduce the secretion of apolipoprotein B100 and lipids, such as very low-density lipoprotein (VLDL) and low-density lipoprotein (LDL) (45). It is known that VLDL is an important transporter in the liver of dairy cows and has the function of transferring triglycerides. The reduction in taurine in the milk whey of dairy cows with IO may hinder VLDL synthesis to make triglycerides deposition in the liver, which causes gluconeogenesis to worsen the negative energy balance and the postpartum estrus in dairy cows. The combination of acetic acid with coenzyme A into acetyl CoA is essential for the metabolism of carbohydrates and fats (46). The BHBA is an incomplete metabolic product of fatty acids in the liver (47). When the blood glucose is too low to supply energy, fat mobilization is enhanced to produce more ketone bodies (48). In this experiment, both acetic acid and BHBA increased in the serum of dairy cows with IO, suggesting that the cows' lipid metabolism was mobilized to meet lactation energy demand due to the lack of glucose. Therefore, high BHBA and low glucose in dairy cows with IO affect the development of follicles due to abnormal amino acid metabolism. Increased levels of BHBA in dairy cow serum indicate that dairy cows may suffer from ketosis or subclinical ketosis. Studies have confirmed that cows suffering from ketosis have an increased probability of anestrus (49).

Inositol is an important component of certain lipids (50). It can combine with choline to form phosphatidylinositol and reduce blood cholesterol (51). Studies have shown that, in follicular membranes and granulosa cells, inositol may maintain steroid production activity and sex hormones during the ovarian cycle by regulating the dynamics of the cytoskeleton structure (52). Inositol is mainly produced from glucose in the muscle (53). The decreased glucose level in the serum of dairy cows with IO reduces the synthesis of inositol, affecting the synthesis of sex hormones.

### Similarities and Differences of Whey Metabolites and Serum Metabolites

The metabolites in milk whey result from the *de novo* synthesis of the serum and mammary glands and are affected by the metabolic state in mammary glands of lactating cows (54). The small molecular metabolites in the milk whey of the mammary glands are composed of precursors in the blood (55, 56). The serum and milk whey components of dairy cows are affected by nutritional intake, disease state, and lactation period, and the specific metabolic relationship between them is limited (25). Ilves et al. found that there seems to be little correlation between the molecular composition of plasma and milk. Compared with milk, blood is more dependent on individual animals, and citrate and lactose have the greatest impact because they are more abundant in milk (57). Maher et al. reported that milk is a unique metabolic system, and its metabolite composition is largely unaffected by plasma composition under normal conditions. However, trimethylamine and dimethyl sulfone are highly correlated in the plasma and milk, and plasma valine levels are related to differences in amino acid catabolism in the mammary gland (25). Our studies also confirmed the inconsistent changes in the differential metabolites in the serum and milk whey of dairy cows with IO. Succinic acid is an intermediate of TCA and plays a key role in ATP production in mitochondria, is an important intermediate of several metabolic pathways, and is involved in the formation and elimination of reactive oxygen species (58). Succinic acid content in the serum of dairy cows with IO is reduced but is increased in the milk whey. The content of inositol level is the same as succinic acid in the serum and milk whey. It is unclear if there may be a special need of the mammary gland in dairy cows with IO during early lactation, which need to be further confirmed in the future.

In summary, this study explored the differences between the serum and milk whey of dairy cows with inactive ovaries postpartum 70 days and found that inactive ovaries in dairy cows are closely associated with decreased glucose metabolism, impaired tricarboxylic acid cycle, increased lipid mobilization, and abnormal amino acid metabolism. More research will be needed to further demonstrate how these differential metabolisms affect postpartum inactive ovaries in dairy cows.

## Data Availability Statement

The datasets presented in this study can be found at the following link: https://www.iprox.org/page/project.html?id=IPX0002687000.

## Ethics Statement

The animal study was reviewed and approved by Animal Welfare and Research Ethics Committee at Heilongjiang Bayi Agricultural University.

## Author Contributions

CZ wrote this article and completed the experiment. YB and SF completed the work of collecting samples from cattle farms. LW mainly completes the data analysis of this experiment. CXi and CXu provided help and guidance for the writing of this article. All authors contributed to the article and approved the submitted version.

## Conflict of Interest

The authors declare that the research was conducted in the absence of any commercial or financial relationships that could be construed as a potential conflict of interest.
